# Supplementary material for: Nonlinear and nonlocal elasticity in coarse-grained differential-tension models of epithelia
Source: Phys Rev E. Author manuscript; Available in PMC 2024 Jun 10. (PMC7616085; doi:10.1103/PhysRevE.99.022411)
Supplement: Appendix [file EMS196592-supplement-Appendix.pdf]

APPENDIX A: DERIVATION OF EQ. (12)

In this Appendix, we relate ϕ and ψ by deriving Eq. (12) used in the main text. At the same time, we verify that this expansion is indeed consistent at all orders. Taylor expanding the right-hand side of Eq. (11),

$$\begin{aligned}\psi(s+ks_0)-\psi(s) &= \phi(s)+2\left(\sum_{j=1}^{k-1}\phi(s+js_0)\right)+\phi(s+ks_0) = 2k\phi(s)+\sum_{n=1}^{\infty}\frac{s_0^n}{n!}\phi^{(n)}(s)\left\{k^n+2\sum_{j=1}^{k-1}j^n\right\}\\ &= 2k\phi(s)+\sum_{n=1}^{\infty}\frac{s_0^n}{n!}\phi^{(n)}(s)\left\{k^n+\frac{2}{n+1}\sum_{j=0}^{n}(-1)^j\binom{n+1}{j}\mathcal{B}_j(k-1)^{n+1-j}\right\},\end{aligned} \tag{A1}$$

where we have used Faulhaber's formula [46] to expand the sum of powers of integers, and where $\mathcal{B}_0=1, \mathcal{B}_1=-\frac{1}{2},\ldots$ denote the Bernoulli numbers (of the first kind) [32]. Expanding $(k-1)^{n+1-j}$ using the binomial theorem and simplifying the binomial coefficients,

$$\psi(s+ks_0)-\psi(s)=2k\phi(s)+\sum_{n=1}^{\infty}\frac{\phi^{(n)}(s)}{n!\ell_0^n}A(k,n), \tag{A2}$$

where we have introduced

$$\begin{aligned}A(k,n) &= k^n+2(-1)^{n+1}n!\sum_{j=0}^{n}\sum_{i=0}^{n+1-j}\frac{(-1)^i\mathcal{B}_jk^i}{i!j!(n+1-i-j)!}\\ &= \sum_{i=0}^{n+1}a_i(n)k^i,\end{aligned} \tag{A3}$$

wherein $a_0, a_1, \ldots, a_{n+1}$ depend on n. In particular,

$$a_0=0,\quad a_n=0,\quad a_{n+1}=\frac{2}{n+1}, \tag{A4}$$

of which the last two are obtained by direct computation, and the first one follows using an identity of Bernoulli numbers [32],

$$\sum_{j=0}^{n}\binom{n+1}{j}\mathcal{B}_j=0\quad\text{for } n=1,2,\ldots. \tag{A5}$$

Moreover, for $i=1,2,\ldots,n-1$,

$$a_i(n)=2(-1)^{n+1-i}\frac{n!}{i!}\sum_{j=0}^{n+1-i}\frac{\mathcal{B}_j}{j!(n+1-i-j)!}. \tag{A6}$$

Accordingly,

$$A(k,n) = \frac{2k^{n+1}}{n+1} + 2(-1)^{n+1}n!\sum_{i=1}^{n-1}(-1)^i\frac{k^i}{i!}\left\{\sum_{j=0}^{n+1-i}\frac{\mathcal{B}_j}{j!(n+1-i-j)!}\right\}. \tag{A7}$$

Upon inverting the order of summation, Eq. (A2) becomes

$$\psi(s+ks_0)-\psi(s) = 2k\phi(s) + \sum_{n=1}^{\infty}\frac{\phi^{(n)}(s)}{n!\ell_0^n}\left(\frac{2k^{n+1}}{n+1}\right) + \sum_{i=1}^{\infty}\sum_{n=i+1}^{\infty}2(-1)^{n+1-i}\frac{\phi^{(n)}(s)}{\ell_0^n}\frac{k^i}{i!}\left\{\sum_{j=0}^{n+1-i}\frac{\mathcal{B}_j}{j!(n+1-i-j)!}\right\}. \tag{A8}$$

Finally, upon relabelling indices in the first summation and changing variables $n \longmapsto m = n+1-i$ in the last summation,

$$\psi(s+ks_0)-\psi(s) = \sum_{i=1}^{\infty}\frac{k^i}{i!\ell_0^i}\left\{2\ell_0\phi^{(i-1)}(s) + \sum_{m=2}^{\infty}\frac{2(-1)^m}{\ell_0^{m-1}}\phi^{(i-1+m)}(s)\left(\sum_{j=0}^{m}\frac{\mathcal{B}_j}{j!(m-j)!}\right)\right\}. \tag{A9}$$

But, rearranging Eq. (A5),

$$\sum_{j=0}^{m}\frac{\mathcal{B}_j}{j!(m-j)!} = \frac{\mathcal{B}_m}{m!} \quad \text{for } m = 2, 3, \ldots. \tag{A10}$$

Since $\mathcal{B}_n = 0$ for odd $n > 1$, and using $\mathcal{B}_0 = 1$, we finally obtain

$$\psi(s+ks_0)-\psi(s) = \sum_{i=1}^{\infty}\frac{k^i}{i!\ell_0^i}\left\{\sum_{m=0}^{\infty}\frac{2\mathcal{B}_{2m}}{(2m)!}\frac{\phi^{(i-1+2m)}(s)}{\ell_0^{2m-1}}\right\}. \tag{A11}$$

Comparing this to the Taylor expansion of the left-hand side,

$$\psi(s+ks_0)-\psi(s) = \sum_{i=1}^{\infty}\frac{k^i}{i!\ell_0^i}\psi^{(i)}(s), \tag{A12}$$

we deduce that the expansion is consistent at all orders, with, in particular,

$$\psi'(s) = \sum_{m=0}^{\infty}\psi_m\frac{\phi^{(2m)}(s)}{\ell_0^{2m-1}} \quad \text{where } \psi_m = \frac{2\mathcal{B}_{2m}}{(2m)!}, \tag{A13}$$

which is Eq. (12). As noted in the main text, we are not aware of a closed form for the inverted series that expresses ϕ as a function of the derivatives of ψ. Formally, inverting Eq. (12) gives

$$\phi(s) = \sum_{m=0}^{\infty}\phi_m\frac{\psi^{(2m+1)}(s)}{\ell_0^{2m+1}}, \tag{A14}$$

where the coefficients $\phi_0, \phi_1, \ldots$ are determined recursively by $\phi_0\psi_0 = 1$ and

$$\sum_{j=0}^{m}\phi_j\psi_{m-j} = 0 \quad \text{for } m = 1, 2, \ldots. \tag{A15}$$

In agreement with Eq. (13), we find

$$\phi_0 = \tfrac{1}{2}, \quad \phi_1 = -\tfrac{1}{24}, \quad \phi_2 = \tfrac{1}{240}, \ldots. \tag{A16}$$

APPENDIX B: EIGENMODES OF THE BUCKLED EPITHELIUM

Eigenmodes of the epithelium are nonzero solutions of the governing Eq. (21) with $\mu = 0$. They thus obey

$$\ddddot{\psi} = 6\Xi^2\ddot{\psi} - 3\Delta\Xi\dot{\psi}\ddot{\psi} + \tfrac{15}{4}\dot{\psi}^2\ddot{\psi}, \tag{B1}$$

subject to

$$\psi(0) = \psi(1) = 0, \qquad \ddot{\psi}(0) = \ddot{\psi}(1) = 0. \tag{B2}$$

To find eigenmodes numerically, we remove the trivial, zero solution by imposing a nonzero compression D and varying this compression until a solution with $\mu = 0$ is found.

Numerically, we obtain eigenmodes if $\Delta \geqslant \Delta_*$, but find no solutions if $\Delta < \Delta_*$, for some value Δ_* depending on Ξ (Fig. 5). Plotting Δ_* against Ξ (Fig. 5, inset) suggests that Δ_* approaches a constant value as Ξ grows large. We observe that the numerical data are well approximated by a

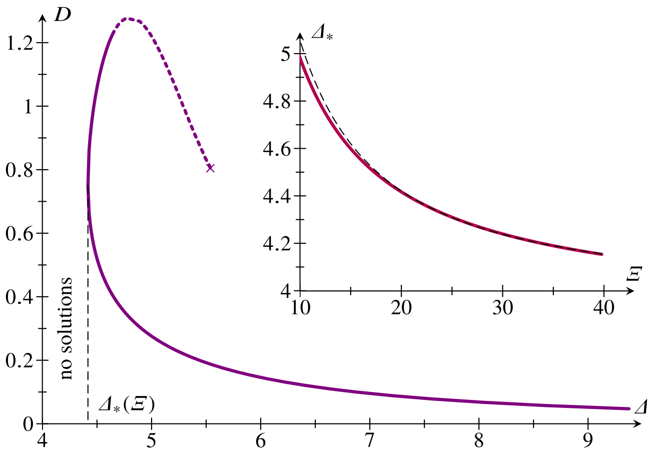

FIG. 5. Eigenmodes of a buckled epithelium: plot of relative end-to-end shortening D against Δ. Parameter value: $\Xi = 20$. No eigenmodes were found for $\Delta < \Delta_*(\Xi)$. On the dashed part of the branch, $\mathcal{E} < 2$. Continuation failed at the point marked $\times$. Inset: plot of Δ_* against Ξ (solid line) and power-law fit (dashed line).

power-law $\Delta_* = c_1 + c_2\,\Xi^{-5/4}$, where $c_1 \approx 3.96$, $c_2 \approx 19.5$ (Fig. 5, inset).

Some of the solutions in Fig. 5 have energy $\mathcal{E} < 2$, lower than the energy of the uncompressed, flat solution; these are spontaneous buckled modes that arise in the absence of external forces, but, as is apparent from the corresponding values $D > 1$ (Fig. 5), these solutions are unphysical. In Ref. [10], the flat configuration of the epithelium becomes unstable at large enough differential tension. This instability, absent in the present description, arises because the analysis of Ref. [10] does not impose the condition that the cells match up exactly along their lateral sides [35].

The "large" values of Δ and hence δ for these eigenmodes beckon a comment on the formal range of validity of the continuum model: stability of the underlying discrete model requires $\alpha, \beta \geqslant 0$ [7], and hence $\delta \leqslant \ell_0^2$. While the asymptotic expansion leading to the geometric relation (13) was an expansion in the large parameter ℓ_0, it did not involve δ. By contrast, the expansion of the Lagrangian (20), which did involve δ, was an expansion in a different large parameter, Ξ. Hence, large values of $\delta \lesssim \ell_0^2$ are indeed in the formal range of validity of the continuum model provided that Ξ is large enough.
